# Editorial: Organic Semiconductors: Investigating the Processing-Structure-Property Relationships

**DOI:** 10.3389/fchem.2021.745170

**Published:** 2021-08-03

**Authors:** Kui Zhao, G. N. Manjunatha Reddy, Yanchun Han

**Affiliations:** ^1^ Shaanxi Normal University, Xi’an, China; ^2^ Univ. Lille, CNRS, Centrale Lille Institut, Univ. Artois, UMR 8181, Unité de Catalyse et Chimie du Solide, Lille, France; ^3^ Changchun Institute of Applied Chemistry, Chinese Academy of Sciences, Changchun, China

**Keywords:** conjugated polymers, morphology, crystallization, solar cells (441), field-effect transistor, organic light-emitting diode

Solution-processed semiconductor materials exhibit promising optical and electronic properties, and found applications in solar energy harvesting, display technologies and bioelectronics. In this context, organic semiconductors (OSC) have garnered intense interest for their integration into a range of optoelectronic devices such as organic light-emitting diodes (OLED), organic photovoltaics (OPV), organic field-effect transistors (OFET), as well as emerging technologies such as wearable electronics. The merits of OSCs for such applications include low synthetic cost, light weight and high scalability of synthesis, facile deposition techniques to achieve, for example, printed electronics, large area and flexible solar cells. Of the aforementioned technologies, OLEDs have reached commercialization in products such as smartphones and displays. OPV technology is beginning to be considered as potentially viable, with power conversion efficiencies (PCEs) of over 18% recently reported in single active layer devices. In addition, indoor OPV (IPOV) technology is gaining momentum owing to the tunable energy levels of organic materials to match the indoor spectrum.

We are pleased to introduce *Frontiers in Chemistry* special issue Research Topic “Organic Semiconductors: Investigating Processing-Structure-Property Relationships”. This collection showcases articles and Reviews on various aspects of OSC research, as well as a current status assessment of its role in state-of-the-art materials design and characterization.

Single molecule or polymeric OSCs are composed of π-conjugated donor and acceptor building blocks. A key parameter to develop OSCs for specific applications is the energy gap between electronic states of π-conjugated building blocks, and adjustability of this energy gap. This can be achieved by chemical design of small molecules, building blocks and polymeric OSCs. The OSC thin film morphology is governed by the compositional and structural heterogeneities associated with these building blocks and deposition techniques such as vapor deposition and solution processing techniques. In addition, molecular self-assembly and solid-state organization of the material determines the overall optoelectronic properties that enable device performance (e.g., charge transport, photoinduced charge generation, and/or optical performance). For example, OSC-based photoactive layer determines the efficiency of charge generation, separation and how the resulting charges transit to the electrodes. A bottleneck challenge is the development of reliable structure-processing-property relationships.

Crystallization process of polymers plays a significant role in determining their structures, degree of order and functional properties. Additives can be added to control the crystal growth in order to tune sizes and shapes of molecular assemblies at different length scales. As part of this Research Topic, Yang et al. showed that large (up to micrometer size) crystals of n-type OSC inks can be grown by adding a p-type semiconductor to a processing solvent. This strategy has been used to regulate the crystallization behavior of N, N′-dioctyl perylene diimide (C8PDI) using poly(3-hexylthiophene) (P3HT) and polyethylene glycol (PEG) additives in an aqueous solution. Specifically, C8PDI crystals of sub-micro to micrometer (0.4–2 μm) length and 35–210 nm width have been synthesized. Using this approach, stable and pollution-free aqueous OSC inks were prepared for large area printing of electronic devices.

Research on organic solar cells is enticing. Central to the performance of organic solar cells is the bulk-heterojunction (BHJ) morphology with optimal donor-acceptor intermix and phase separation, which contributes to the overall optoelectronic properties. The advent of non-fullerene acceptors (NFAs) and high efficiency donor polymers enables high PCEs of over 18% to be achieved in single-junction solar cells. There is a great current interest in the development of NFAs for BHJ based organic solar cells. Liang et al., showed that the fluorination of IDIC NFA end groups enhance the miscibility when mixed with polythiophene based donor polymers, which boosts the PCE values of solar cells. With the increased number of fluorine atoms in IDIC, the resulting NFAs showed enhanced miscibility with PDCBT-Cl donor polymer, and substantially reduced the domain sizes in the BHJ blend films. A multitechnique characterization approach utilizing calorimetry, electron microscopy, and X-ray scattering was used to establish a relationship between the number of fluorine atoms, miscibility, and device performance in PDCBT-Cl:IDIC solar cells.

There is a growing interest in the pursuit of OSC materials and their blends for indoor photovoltaics applications. In an article by Bi et al., BHJ blends based on poly(thiophene vinylene) (PTV) donor polymers with ITCC NFAs have been used to formulate indoor photovoltaic cells, which showed PCEs of 9.6% (AM 1.5 G sunlight) and 24.27% (under 1,000 lux, LED 2,700 K), respectively. These materials exhibited preferred orientation of the crystallites, changing from edge-on to face-on alignment, upon replacing the ethylene groups in the backbones of PTVT-V by thiophene moieties in that of PTVT-T, indicating the structure dependent optoelectronic properties in IOPVs. Notably, PTVT-T:ITCC BHJ blends exhibited lower energetic disorder (58 meV) than the PTVT-V:ITCC blend (70 meV), leading to lower recombination rates and enhanced charge transport. These results indicate that the chemical design of PTV-based polymers would further enhance the photovoltaic properties by suppressing the energetic disorder, which is a promising way to realize low-cost IOPV cells.

Optical sensors based on OSCs is an area of research that is gaining momentum. Given the wider applications of circularly polarized light (CPL) in the areas of photonics, integrated photoelectric sensors capable of directly detecting CPL are essential to develop photonic devices. For example, photodetectors based on chiral OSCs that can directly detect CPL would further enhance photonics applications. A Review by Zhang et al. summarizes the recent advancements in CPL photodetectors based on chiral OSC including the working principles of CPL photodetectors, current challenges and future opportunities in the field. The active materials for such applications range from the chiral enantiomer 1-aza[6]helicene synthesized by complex methods in 2013 to chiral structure metamaterials in 2015 and to chiral organic-inorganic perovskite materials in 2019. Furthermore, the methods to prepare photosensitive CPL materials and the detector performance have been greatly improved, for example, the sensing region of CPL detectors based on chiral nanostructured materials has been extended to 1,340 nm, and the responsivity (R) has been improved to 300 A W^−1^. The anisotropy factor (gres) of CPL photodetectors has increased to 1.9, demonstrating excellent distinguish ability between LCP and RCP photons. These improvements in device performance and preparation methods play a very important role in the practical applications of CPL photodetectors.

This special issue thus represents diverse aspects ranging from state-of-the-art synthesis, characterization, device physics and applications of OSCs in the emerging areas such as solar cells and photodetectors. We are indebted to the authors for their excellent contributions in making up this Research Topic, and thankful to *Frontiers in Chemistry* for their editorial support. We envision that the concepts presented in this special issue will stimulate further discussions and fundamental understandings of the processing-structure-property relationships in OSCs, and reach the broad readership of *Frontiers in Chemistry.*

